# Emodin-Loaded Magnesium Silicate Hollow Nanocarriers for Anti-Angiogenesis Treatment through Inhibiting VEGF

**DOI:** 10.3390/ijms150916936

**Published:** 2014-09-23

**Authors:** Hua Ren, Chao Zhu, Zhaohui Li, Wei Yang, E Song

**Affiliations:** 1 Department of Ophthalmology, First Hospital of Jilin University, Changchun 130021, China; E-Mails: jlurenhua@yeah.net (H.R.); jluyangwei@yeah.net (W.Y.); 2 Department of Ophthalmology, Second Hospital of Jilin University, Changchun 130021, China; E-Mail: jluzhuchao@yeah.net; 3 Department of Ophthalmology, People’s Hospital of Changchun City, Changchun 130021, China; E-Mail: jlulizhaohui@yeah.net

**Keywords:** nanoparticle, emodin, VEGF (Vascular endothelial growth factor)

## Abstract

The applications of anti-VEGF (vascular endothelial growth factor) treatment in ophthalmic fields to inhibit angiogenesis have been widely documented in recent years. However, the hydrophobic nature of many agents makes its delivery difficult in practice. Therefore, the aim of the present study was to introduce a new kind of hydrophobic drug carrier by employing nanoparticles with a hollow structure inside. Followed by the synthesis and characterization of magnesium silicate hollow spheres, cytotoxicity was evaluated in retina capillary endothelial cells. The loading and releasing capacity were tested by employing emodin, and the effect on VEGF expression was performed at the gene and protein level. Finally, an investigation on angiogenesis was carried on fertilized chicken eggs. The results indicated that the magnesium silicate nanoparticles had low toxicity. Emodin–MgSiO_3_ can inhibit the expression of both VEGF gene and protein effectively. Angiogenesis of eggs was also reduced significantly. Based on the above results, we concluded that magnesium silicate hollow spheres were good candidates as drug carriers with enough safety.

## 1. Introduction

Ocular neovascularization (NV) is a main cause of blindness associated with ischemic retinal disorders, including proliferative diabetic retinopathy (PDR), retinopathy of prematurity (ROP) and age-related macular degeneration (AMD) [1,2]. Retinal neovascularization is largely the result of angiogenesis, in which cells of endothelial origin from the existing blood vessels start to proliferate out of control [3]. Although a lot of energy and effort have been paid to clarifying the molecular mechanism involved, few breakthroughs have been acquired in the past few decades [4]. The exact mechanism underlying the pathogenesis remains unknown; hence, there is no satisfactory therapy.

Vascular endothelial growth factor (VEGF) is the most potent cytokine that stimulates angiogenesis in retina [5,6]. Among the five subtypes, VEGF-A has more affinity for angiogenic tissue. VEGF-A realizes its functions through binding to vascular endothelium expressing receptors VEGFR-1 (tyrosine kinase receptor) and VEGFR-2 (KDR/kinase insert domain-containing receptor) [7]. It has been reported that injection of exogenous VEGF to the eyes of monkeys can cause NV of retina [8,9]. On the other side, the application of anti-VEGF antibodies was proven to alleviate the severity of NV [10]. Up to now, a great number of agents aimed at anti-VEGF have been recorded, and this has been regarded as one of the most effective strategies [11,12].

Emodin is an anthraquinone derivative from the rhizome of *Rheum palmatum* L., a plant widely used in traditional Chinese medicine as a laxative [13,14]. Previous research showed that emodin possesses antifungal, antibacterial, antiviral and anti-VEGF activities [15]. It was shown that emodin inhibits endothelial cell proliferation and has different effects on endothelial and tumor cell angiogenesis. In the context of the eye, emodin lessened inflammation and scarring in a mouse ocular alkali burn model [16], so it is regarded as a promising candidate for ocular neovascularization. However, like many other anti-VEGF agents, the application of emodin is limited due to its hydrophobicity. Therefore, it is important and necessary to discover new methods for efficient delivery.

In recent years, the development of nanomaterials has drawn considerable attention for its diverse applications. One of these is the construction of a drug delivery system [17] that can exceedingly reduce harmful nonspecific side-effects and toxicity compared to other types of carriers. More importantly, nanocarriers can commonly overcome low concentrations of drugs and modify the characteristic of the drug [18]. Due to the large fraction of voids in their inner space, hollow micro- and nano-structures have emerged as powerful candidates in a wide range of applications, including drug delivery, efficient catalysis, waste removal, gas sensors, as well as lithium-ion batteries [19,20]. In particular, the use of silica-based nanoparticles has shown ever-increasing advances in biocompatibility and has achieved great success in the fields of bioengineering [21,22]. Therefore, the aim of the current research is to construct a system by employing hollow nanoparticles as the carriers for better emodin delivery.

## 2. Results and Discussion

Known as angiogenesis, the development of new blood vessels from preexisting vasculature has a causal role in ocular neovascularization. VEGF plays the role of a positive regulator of angiogenesis, and its proven role in angiogenesis has provided evidence for the use of anti-VEGF agents as potential therapies [23]. As a result, VEGF inhibitors have been applied as the mainstay in the treatment of ocular neovascularization. Most anti-VEGF drugs for ocular disease treatment are applied as eye drop formulations or invasive injections. The former drug delivery system is regarded as low efficiency, and the latter one usually brings fear to patients. Furthermore, the concentration of drug in retina hardly turns out to be effective, because of ocular barriers [24]. Therefore, it is meaningful to invite a new system for efficient and comfortable drug delivery. Up to now, nanoparticles have been regarded as a new approach to achieve this goal. In the present study, we showed that hollow MgSiO_3_ nanoparticles are potent carriers of emodin and inhibit the function of retinal capillary endothelial cells *in vitro*.

As described previously, the formation of SiO_2_ colloidal spheres in this system heavily depends on the classical Stöber method [25]. Then, the facile hydrothermal approach was used to acquire the growing of MgSiO_3_ hollow structures on the basis of a chemical-template etching mechanism. Specifically, silica colloidal spheres were incubated with an alkaline solution, which contains magnesium ions, ammonium ions and ammonia. Then, the solution was transferred to a Teflon-lined stainless-steel autoclave. Through high-temperature treatment, the silica chains were broken by hydroxide ions, and silicate-ion groups were generated. These silicate ions released from SiO_2_ colloidal spheres react with magnesium ions, and magnesium silicate was constructed *in situ* around the SiO_2_ cores. Followed by the slow release of silicate ions from the SiO_2_ cores, well-structured magnesium silicate hollow spheres are formed gradually.

**Figure 1:**
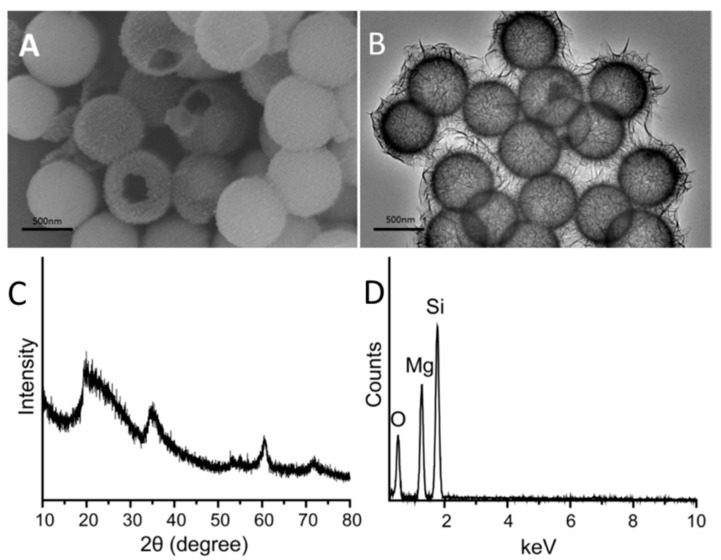
(**A**) Scanning electron microscope (SEM) image; (**B**) transmission electron microscope (TEM) images of MgSiO_3_ nanoparticles; (**C**) wide-angle XRD (X-ray diffraction) pattern of uniform MgSiO_3_ hollow spheres; and (**D**) the EDS (energy-dispersive spectroscopy) spectrum of uniform MgSiO_3_ hollow spheres.

The morphological and structural analyses of the MgSiO_3_ hollow spheres were carried via scanning electron microscopy (SEM) and transmission electron microscopy (TEM) at first. As exhibited in Figure 1A,B, MgSiO_3_ hollow spheres with a uniform and divergent nature revealed a relatively coarse surface and a mean diameter of ~400 nm. Since the average diameter of the SiO_2_ precursor we used was 380 nm, the average diameter of our sample is nearly 400 nm. TEM image shown in Figure 1B indicates that the magnesium silicate spheres possess a hollow structure with an obviously darkish center. Furthermore, the edge is black, and thickness of the shell of the hollow structures was about 30 nm. Actually, needle-like structures could be observed at first glance through the SEM and TEM images. The hollow structures were constituted of large, narrow and nanoscale lamellae. Phase purity and crystalline order are two of the most important parameters affecting the properties of materials. Wide-angle x-ray diffraction (XRD) patterns shown in Figure 1C demonstrate that all of the well-defined diffraction peaks present the features of MgSiO_3_. Energy-dispersive spectroscopy (EDS) analysis further confirmed that our product consists of magnesium, silicon and oxygen elements (Figure 1D).

**Figure 2:**
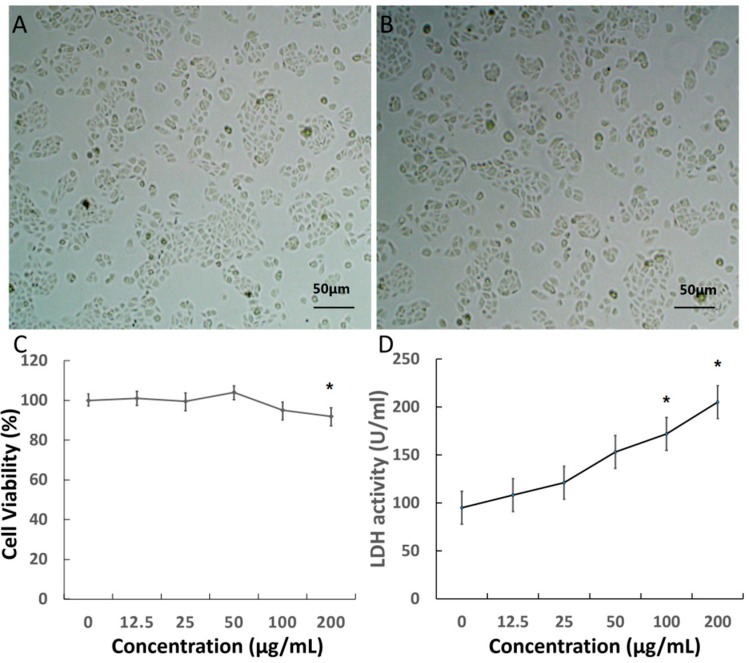
(**A**,**B**) Visible light microscopy images of retinal capillary endothelial cells incubated with nanoparticles and control; (**C**) the effect of MgSiO_3_ hollow spheres on the viability of cells; and (**D**) LDH (lactate dehydrogenase) activity induced by MgSiO_3_ nanoparticles. (* *p* < 0.05).

Prior to using nanomaterials for bio-systems applications, we investigated the cytotoxicity of the MgSiO_3_ hollow spheres. The MTT assay associated with retina capillary endothelial cells was applied to evaluate the cytotoxicity of MgSiO_3_ hollow spheres. We investigated the behavior of the MgSiO_3_ hollow spheres in living cells via observing the morphological changes of cells using a microscope. As shown in Figure 2A,B, microscopy images illustrated no obvious difference in the cell morphology for the cells treated with hollow spheres compared to the control groups after incubation with nanoparticles. As illustrated in Figure 2C, cell viability was not hindered by nanoparticles up to a concentration of 200 μg/mL after 48 h of incubation. Otherwise, lactate dehydrogenase (LDH) activity induced by nanoparticles at various concentrations was also tested to evaluate cytotoxicity. This obviously shows that LDH activity became significant until the concentration was higher than 100 μg/mL (Figure 2D), thereby indicating satisfactory results that supported the biocompatibility of the hollow spheres in the applied dosages.

**Figure 3:**
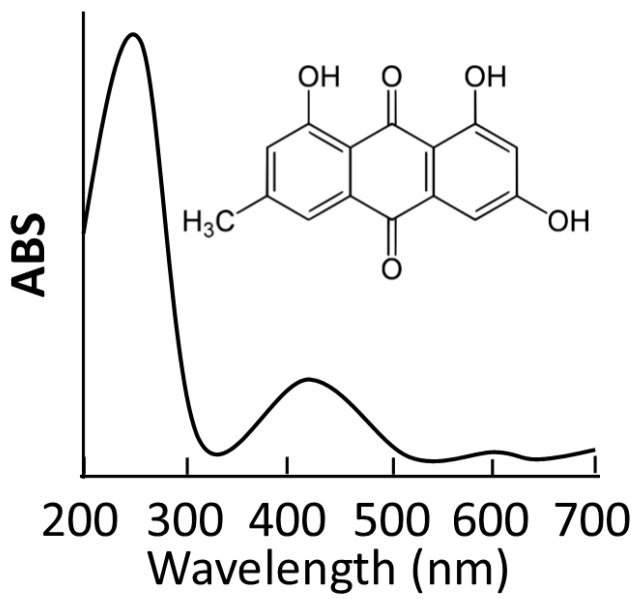
UV-Vis spectrum and chemical structure of the emodin molecules.

Up to now, the lack of aqueous solubility has always limited practical application for anti-VEGF treatment in clinics. To overcome this bottleneck, hollow materials have held more potential for the development of novel delivery systems [25,26]. These are characterized by a large space for storing guest molecules, which is an excellent feature for efficient nano-carriers. Emodin was employed as a model of a hydrophobic anti-VEGF drug in our current study. The MgSiO_3_ hollow spheres were loaded with emodin molecules by soaking them in dimethyl sulfoxide (DMSO) containing emodin for 24 h. After emodin–MgSiO_3_ was removed via careful centrifugation, emodin–MgSiO_3_ was washed twice with phosphate buffer saline (PBS) to remove additional adsorbed drug on the surface of the MgSiO_3_ hollow spheres. Detected via UV-Vis spectroscopy, approximately 130 μg of the emodin molecules were stored inside 1 mg of the hollow spheres, demonstrating the high loading capacity of the MgSiO_3_ hollow spheres (Figure 3).

**Figure 4:**
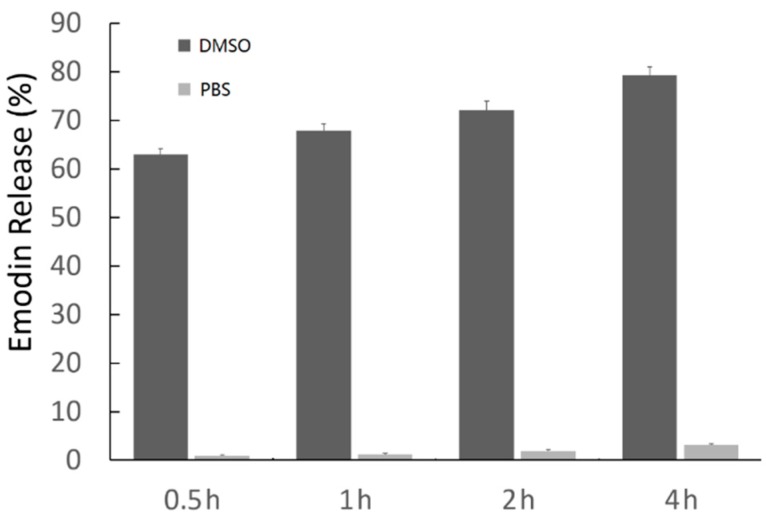
The amounts of emodin released from the emodin–MgSiO_3_ complex measured via UV-Vis spectroscopy.

**Figure 5:**
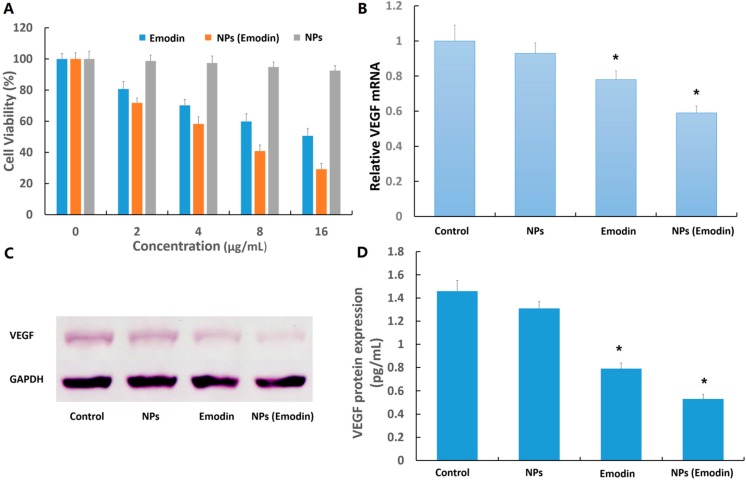
(**A**) Viability of cells incubated with emodin and emodin–MgSiO_3_ under different incubation concentrations; (**B**) VEGF (vascular endothelial growth factor) mRNA expression of cells; (**C**) VEGF protein expression measured by western blot; and (**D**) enzyme-linked immunosorbent assay (ELISA) analysis for VEGF protein expression.(* *p* < 0.05).

Figure 4 shows the amount of emodin released when emodin–MgSiO_3_ was dispersed in PBS (pH = 7.4) and DMSO with different incubation periods. Less than 4% of the stored emodin could be released into the supernatant when emodin–MgSiO_3_ was dispersed in PBS and left in suspension for up to 4 h. However, once emodin–MgSiO_3_ was dispersed in DMSO for 0.5 h, most of the emodin could be released and detected in the supernatant. The above results suggest that the negligible drug leakage of emodin–MgSiO_3_ in PBS has great significance in the minimization of side effects. The viabilities of retina capillary endothelial cells against emodin and emodin–MgSiO_3_ after incubation for 24 h are present in Figure 5A. Compared with the control group, both emodin and emodin–MgSiO_3_ exhibited toxicity towards retina capillary endothelial cells. The slight cytotoxicity of free emodin was probably in accordance with the low solubility of emodin in the hydrophilic medium. The enhanced cytotoxicity of emodin–MgSiO_3_ further made out the efficient drug-delivery capability by using carriers. Due to the presence of hydrophobic sections in the cells, emodin–MgSiO_3_ could rationally release emodin at these active sites, which plays an important role in inhibiting the proliferation of cells.

**Figure 6:**
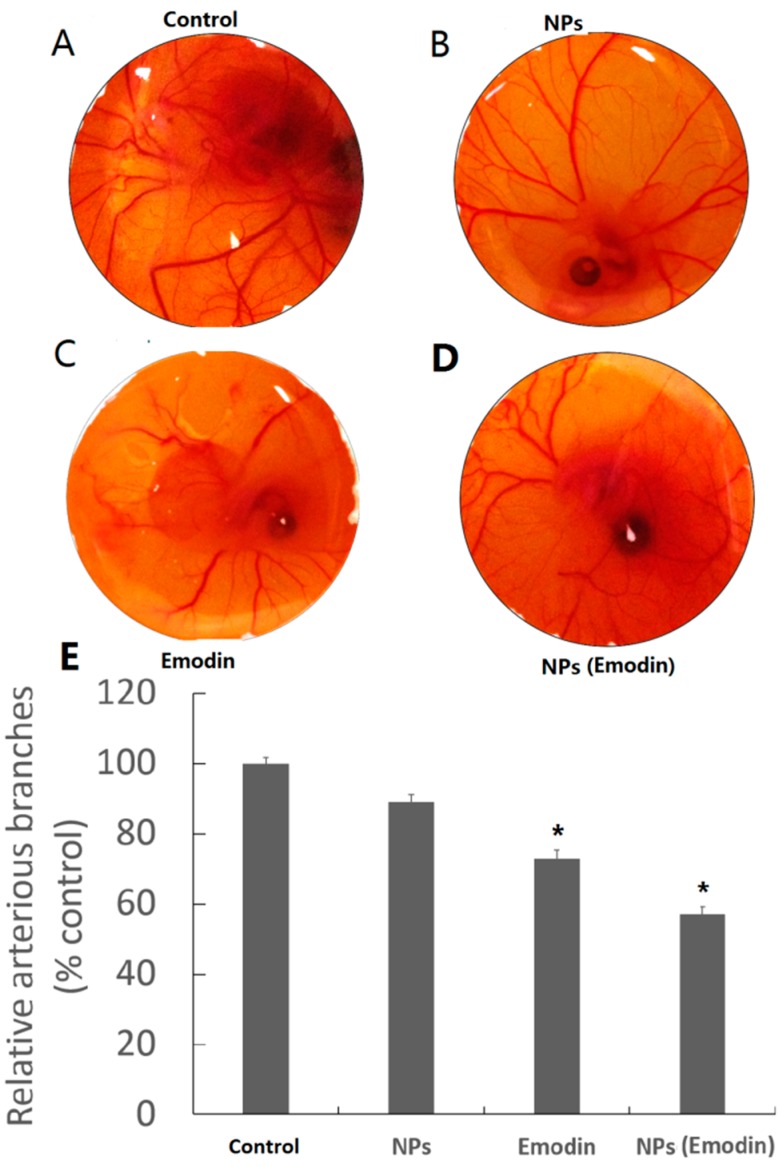
Blood vessel formation of fertilized chicken eggs incubated with different agents (**A**–**D**) and quantitative analysis (**E**). (* *p* < 0.05).

To evaluate emodin and emodin–MgSiO_3_ hollow spheres for *in vitro* anti-VEGF therapy, the expression of VEGF gene and protein was studied. Transcripts for VEGF in the presence of emodin and emodin–MgSiO_3_ were measured after 24 h of incubation. As shown in Figure 5B, the presence of free emodin could downregulate the expression in accordance with a previous report [27]. Otherwise, VEGF mRNA expression was significantly inhibited when incorporated with emodin–MgSiO_3_, suggesting the effectiveness of our nano-carrier. Furthermore, we examined the expression of VEGF protein by two methods. As a critical agent on angiogenesis, VEGF is well known for its distinct effect on capillary endothelial cell proliferation and action. As shown in Figure 5C,D, VEGF expression induced by emodin and emodin–MgSiO_3_ is significantly downregulated, while no change is present in the presence of single MgSiO_3_ nanoparticles. To demonstrate the robust features of emodin-loaded MgSiO_3_ hollow spheres, the *in vivo* inhibitory effect on angiogenesis was then demonstrated on fertilized chicken eggs. Blood vessel formation was compared among a single dose of PBS, free MgSiO_3_ nanoparticles, free emodin, as well as emodin-loaded nanoparticles, respectively. Illustrated by Figure 6, less and slenderer vessels were observed in the presence of emodin and emodin–MgSiO_3_, indicating the validity of our nanoparticles.

**Figure 7:**
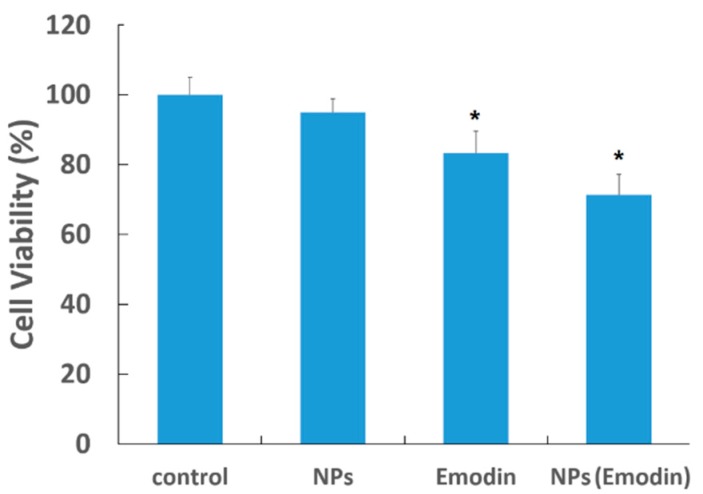
Viability of HUVECs incubated with emodin and emodin–MgSiO_3_. (* *p* < 0.05).

Since the anti-angiogenesis effect of single emodin or emodin–MgSiO_3_ has been proven in retinal capillary endothelial cells, here we further tested the cytotoxicity of our emodin-loaded nanoparticles by employing human umbilical vein endothelial cells (HUVECs). As presented in Figure 7, a similar inhibitory effect on proliferation was observed in HUVECs treated by emodin or emodin–MgSiO_3_, while single nanoparticles (NPs) were less effect. This result raised the potential application of our nanoparticles in the treatment of other angiogenesis-related diseases, but not ocular neovascularization only. However, more studies, like cell migration and *in vitro* tube formation, which were not carried out in current research, are still needed to clarify before further applications.

## 3. Experimental Section

### 3.1. Chemicals

Magnesium chloride hexahydrate (MgCl_2_·6H_2_O), ammonia chloride, ammonium hydroxide aqueous solution (28%) and ethanol were purchased from Beijing Chemicals (Beijing, China). Tetraethyl orthosilicate (TEOS) and (3-aminopropyl) trimethoxysilane (APTES) were purchased from Sigma-Aldrich (Shanghai, China). All of the chemicals were applied as received without further purification.

### 3.2. Synthesis of Uniform Magnesium Silicate Hollow Spheres

Monodispersed silica colloidal spheres with an average diameter of 380 nm were synthesized in accordance with the Stöber method. Magnesium chloride (0.75 mmoL), ammonia chloride (10 mmoL), and ammonia solution (1 mL, 28%) were dissolved in deionized water (20 mL). Silica colloidal spheres (100 mg) were dispersed homogeneously in deionized water (20 mL). These two solutions were mixed until homogeneous and then transferred into a Teflon-lined stainless-steel autoclave (50 mL) and sealed to heat at 160 °C. After reaction for 12 h, the autoclave was cooled to ambient temperature naturally. The obtained magnesium silicate hollow spheres (denoted as NPs) were washed with deionized water and ethanol in sequence and then dried in vacuum at 60 °C overnight.

### 3.3. Cell Cultures

Retina capillary endothelial cells were supplied by American Type Culture Collection. Retina capillary endothelial cells were cultured in dulbecco’s modified eagle medium (DMEM) supplemented with 10% fetal calf serum. The culture media contained 100 UI/mL penicillin and 100 UI/mL streptomycin. The human umbilical vein endothelial cells (HUVECs) were cultured in M199 media (Gibco, Cleveland, TN, USA) containing 10% fetal bovine serum (Gibco), 2 mM glutamine (Gibco) and 1% penicillin/streptomycin (Sigma, Shanghai, China). All of the two cell lines were cultured in a humidified atmosphere containing 5% CO_2_ at 37 °C. Cells were harvested by the use of trypsin and were re-suspended in fresh complete medium before plating.

### 3.4. In Vitro Cytotoxicity Assay of NPs

MTT reduction assays were carried out to quantify the cytotoxicity of MgSiO_3_ hollow spheres. In a typical procedure, retina capillary endothelial cells were cultured in 96-well plates at a density of 5000 per well for 12 h to allow the cells to attach. After cell attachment, the media were changed to α-MEM with MgSiO_3_ at the indicated concentrations. After 48 h incubation, 10 μL MTT (5 g/L) were added to each well and cultured for another 4 h. Then, the supernatant was removed, and 150 μL DMSO were added. This was shaken for 15 min for crystal dissolution. The absorbance at 570 nm was measured with a micro-ELISA reader (Synergy2, Biotek, Winooski, VT, USA). The proliferation rate was calculated and compared to the control group.

### 3.5. Lactate Dehydrogenase (LDH) Release Assay

After the exposure of cells to MgSiO_3_ hollow spheres for 24 h, 50 μL of cell culture media were used to detect LDH activity by the LDH kit (Jiancheng, Nanjing, China), according to the manufacturer’s instruction. Cells incubated with 0.25% Triton for 15 min served as the positive control. The absorbance at 440 nm was measured by the Beckman DU-640B UV-visible spectrophotometer (Beckman Coulter Company, Changchun, China).

### 3.6. Emodin Releasing

The release of emodin under different solvent conditions was performed *in vitro*. In detail, 1 mg of emodin–MgSiO_3_ was re-suspended in a centrifuge tube (10 mL) containing 5 mL PBS or DMSO. At certain time intervals, the MgSiO_3_ hollow spheres were centrifuged, and 0.5 mL of release medium containing the free drug were transferred out to determine the concentration of emodin.

### 3.7. In Vitro Cytotoxicity Assays of Free Emodin and Emodin-Loaded NPs

In a typical procedure, retina capillary endothelial cells or HUVECs were cultured in 96-well plates at a density of 5000 per well for 12 h to allow the cells to attach. Subsequently, free emodin and emodin-loaded NPs with the same drug amount were added to the culture medium. After incubation for 8 h, the medium-containing nanoparticles were removed and fresh medium was placed. At the end of the incubation time (48 h), cell samples were treated with MTT for another 4 h, which was followed by the addition of dimethyl sulfoxide (DMSO) to dissolve the formazan crystals. A Bio-Rad model-680 microplate reader (Bio-Rad, Changchun, China) was applied to measure the absorbance at a wavelength of 570 nm.

### 3.8. Real Time PCR Analysis

In detail, cells were seeded in a 24-well plate at 10^4^/well to determine gene expression. Free emodin and emodin-loaded NPs were added to the culture medium. After 8 h, the medium was replaced by fresh medium. Followed by another 72 h-culture, total RNA was isolated using EASY spin Plus (BLKW, Beijing, China), according to the manufacturer’s instructions. RNA was quantified spectrophotometrically at 260 nm (Nanodrop ND1000, Wilmington, DE, USA), and the quality was examined using the ratio of absorption at 260 and 280 nm, with a ratio between 1.9 and 2.1 as acceptable. Reverse transcription was performed with 2 μg of total RNA using PrimeScript RT Reagent Kit (Takara Co., Otsu, Japan). Each sample was then analyzed by quantitative real-time PCR (qPCR) (Stratagene MX3000P, La Jolla, CA, USA) in the SYBR Premix Ex TapII (Takara Co.), setting the cycles as follows: 10 s/95 °C PCR initial activation step; 40 cycles of denaturation for 20 s/95 °C and annealing step for 20 s/60 °C. The change in VEGF mRNA levels was determined by the formula 2^−ΔΔ*C*t^, where Δ*C*_t_ is the value from the threshold cycle (*C*_t_) of the treated sample subtracted from the *C*_t_ value of the untreated or zero time-point control sample. The relative amount of mRNA in the sample was normalized to GAPDH mRNA. VEGF primers (Invitrogen, Carlsbad, CA, USA) used in this research are as follows: forward primer GAGCAAGACAAGAAAATCCC-3, reverse primer CCTCGGCTTGTCACATCTG.

### 3.9. Western Blot

After incubation with free emodin and emodin-loaded NPs for 72 h, 40 μg of total protein were separated by sodium dodecyl sulfate polyacrylamide gel electrophoresis (SDS-PAGE), using a gradient gel ((10%–12%), Bio-Rad Laboratories), transferred to nitrocellulose membrane and analyzed by immunoblotting using chemiluminescence (Santa Cruz, CA, USA). The primary antibodies used were VEGF-A (Abcam, MA, USA, 1:500) or GAPDH (Santa Cruz, CA, USA, 1:1000) and peroxidase-conjugated anti-mouse IgG (Santa Cruz, CA, USA, 1:1000).

### 3.10. ELISA Analysis for VEGF Expression

Cells were cultured under the indicated condition for 24 h. Culture medium was collected and prepared for VEGF ELISA assay. The assays were performed according to the protocol of the manufacturer using a specific ELISA kit (BlueGene Biotech, Shanghai, China). Each protein sample was analyzed in triplicate with parallel 3-well culture plates to ensure accurate results.

### 3.11. Angiogenesis Assay in Fertilized Chicken Eggs

The effect of free emodin and emodin-loaded NPs on *ex vivo* angiogenesis was determined by the angiogenesis assay. Briefly, fertile leghorn chicken eggs were candled on embryonic Day 8; a small opening was made at the top of the live eggs. Different types of emodin for treatment were mixed with 0.5% methyl cellulose in water and gently placed on the egg. The eggs were incubated for 48 h and photographed. The antiangiogenic analysis was indicated by the relative numbers of arterious branches. The assay was performed three times to ensure reproducibility.

### 3.12. Statistical Analysis

All experiments were performed thrice, and the data are expressed as the means ± SD. The difference between mean values was evaluated by using the ANOVA and considered to be statistically significant when *p* < 0.05.

## 4. Conclusions

In summary, we reported the synthesis of magnesium silicate hollow spheres via a facile template-etching route, which processed uniformly-sized, large void spaces, as well as mesoporous shells. These well-prepared MgSiO_3_ hollow spheres exhibited a significantly high storage capacity of drug and held a sustained release property, showing more potential for drug delivery. Cell cytotoxicity tests against PBS, free MgSiO_3_ nanoparticles, free emodin and emodin-loaded nanoparticles demonstrated the high biocompatibility of free nanoparticles and the enhanced cytotoxicity of emodin-loaded nanoparticles compared with free emodin. Based on the above results, *in vitro* investigation of anti-angiogenesis was primarily studied. Emodin-loaded nanoparticles could effectively suppress vein growth by inhibiting VEGF at the gene and protein level. Taken together, nanoparticles based on our well-designed MgSiO_3_ hollow spheres could serve as practical and powerful carriers of anti-angiogenesis drugs, meeting the criteria as a high-performance biomedical material.
